# Isolation and Characterization of *Dehalobacter* sp. Strain TeCB1 Including Identification of TcbA: A Novel Tetra- and Trichlorobenzene Reductive Dehalogenase

**DOI:** 10.3389/fmicb.2017.00558

**Published:** 2017-04-04

**Authors:** Ricardo Alfán-Guzmán, Haluk Ertan, Mike Manefield, Matthew Lee

**Affiliations:** ^1^School of Biotechnology and Biomolecular Sciences, University of New South Wales, SydneyNSW, Australia; ^2^Department of Molecular Biology and Genetics, Istanbul UniversityIstanbul, Turkey

**Keywords:** 1, 2, 4, 5-tetrachlorobenzene, 1, 2, 4-trichlorobenzene, *Dehalobacter*, bioremediation, organohalide respiration, reductive dehalogenase characterization

## Abstract

*Dehalobacter* sp. strain TeCB1 was isolated from groundwater near Sydney, Australia, that is polluted with a range of organochlorines. The isolated strain is able to grow by reductive dechlorination of 1,2,4,5-tetrachlorobenzene to 1,3- and 1,4-dichlorobenzene with 1,2,4-trichlorobenzene being the intermediate daughter product. Transient production of 1,2-dichlorobenzene was detected with subsequent conversion to monochlorobenzene. The dehalogenation capability of strain TeCB1 to respire 23 alternative organochlorines was examined and shown to be limited to the use of 1,2,4,5-tetrachlorobenzene and 1,2,4-trichlorobenzene. Growth on 1,2,4-trichlorobenzene resulted in the production of predominantly 1,3- and 1,4-dichlorobenzene. The inability of strain TeCB1 to grow on 1,2-dichlorobenzene indicated that the production of monochlorobenzene during growth on 1,2,4,5-tetarchlorobezene was cometabolic. The annotated genome of strain TeCB1 contained only one detectable 16S rRNA gene copy and genes for 23 full-length and one truncated Reductive Dehalogenase (RDase) homologs, five unique to strain TeCB1. Identification and functional characterization of the 1,2,4,5-tetrachlorobenzene and 1,2,4-trichlorobenzene RDase (TcbA) was achieved using native-PAGE coupled with liquid chromatography tandem mass spectrometry. Interestingly, TcbA showed higher amino acid identity with tetrachloroethene reductases PceA (95% identity) from *Dehalobacter restrictus* PER-K23 and *Desulfitobacterium hafniense* Y51 than with the only other chlorinated benzene reductase [i.e., CbrA (30% identity)] functionally characterized to date.

## Introduction

Water and soil contamination with chlorinated benzene industrial intermediates and solvents is wide spread (Field and Sierra-Alvarez, 2008). In particular, 1,2,4,5-tetrachlorobenzene (1,2,4,5-TeCB) has been used as a chemical precursor in the production of herbicides, insecticides, and defoliants like 2,4,5-trichlorophenol and 2,4,5-trichlorophenoxyacetic acid (US-EPA Fact Sheet^1^). As with many other hazardous chemicals 1,2,4,5-TeCB reaches the environment through poor handling disposal practices. For example, in China it was shown that 1,2,4,5-TeCB was present in more than 37 wastewater treatment plants in 23 cities and 17 soils near chemical plants, together with other chlorinated compounds like hexachlorobutadiene (HCBD) and hexachlorobenzene (HCB) (Zhang et al., 2014). In Europe and the USA traces of 1,2,4,5-TeCB have been detected in crops such as potatoes and lettuce as well as in rainbow trout (Wang et al., 1995; Stringer and Johnston, 2001). Acute exposure to 1,2,4,5-TeCB may lead to skin, liver, and kidney damage in humans.

Chlorinated benzenes can be biodegraded under anaerobic conditions by organohalide respiring bacteria (OHRB) that use either aliphatic or aromatic organohalides as terminal electron acceptors (Field and Sierra-Alvarez, 2008). *Dehalococcoides mccartyi* strain CBDB1 was the first OHRB isolated able to respire HCB (Jayachandran et al., 2003). Recently, three *Dehalobacter* strains were identified that are able to reductively dechlorinate several chlorinated benzene congeners (Nelson et al., 2014). Even though both genera are able to respire chlorinated benzenes, their dechlorinating capabilities differ. *D. mccartyi* strain CBDB1 is able to remove doubly and singly flanked chlorines (e.g., 1,2,4-TCB to 1,4-DCB), while *Dehalobacter* sp. strain 13DCB1 is able to dechlorinate un-flanked chlorines (e.g., 1,3-DCB to MCB) (Adrian et al., 2000; Nelson et al., 2014).

Organohalide respiring bacteria posses a unique group of membrane-bound, cobalamin-dependent, oxidoreductases known as Reductive Dehalogenases (RDase) (Payne et al., 2015), responsible for the reductive dechlorination of organohalides. OHRB harbor multiple RDase gene homologs (*rdh*) in their genomes. Rdh genes are normally clustered in an operon encoding different parts of the RDases system (*rhdABCT*); *rdhA* encodes for the catalytic subunit of the enzyme, *rdhB* is the membrane-anchoring protein for RdhA. *rdhC* is believed to be a transcriptional regulator for RdhA and *rhdT* encodes for a chaperone protein for the proper folding of RdhA and its exportation to the periplasm through the TAT secretion pathway (Jugder et al., 2015).

All RdhA share common characteristics, as monomers, their size ranges between 45 and 65 kDa, each RdhA contains a single cobalamin per mole of enzyme. It has been recently demonstrated explicitly that the cobalt atom in the cobalamin catalyzes the reduction of the halogen in the substrate (Payne et al., 2015). Two Fe-S clusters are present along with a twin-arginine (TAT) motif (RRXFXK) at the N-terminus that is recognized by RdhT for folding and exportation (Rupakula et al., 2013; Jugder et al., 2015).

To date, CbrA from *D. mccartyi* strain CBDB1 is the only RdhA identified that is able to catalyze the reductive dechlorination of chlorobenzenes. CbrA reduces 1,2,3,4-tetrachlorobenzene and 1,2,3-TCB to 1,2,4-TCB and 1,3-DCB, respectively (Adrian et al., 2007).

In the current study the isolation and characterization of a novel *Dehalobacter* strain able to respire 1,2,4,5-TeCB and 1,2,4-TCB is described, including the functional characterization of TcbA, the RdhA responsible for the reductive dechlorination of both compounds.

## Materials and Methods

### Chemicals

All chemicals were purchased from Sigma–Aldrich (Australia).

### Enrichment Culturing

Enrichments were carried out using bicarbonate buffered minimum salt medium containing (in g/L): 2.5 NaHCO_3_, 1 NaCl, 0.5 MgCl^.^6H_2_O, 0.2 KH_2_PO_4_, 0.3 NH_4_Cl, 0.3 KCl, 0.15 CaCl_2_^.^2H_2_O, 1 ml trace element solution (1000×), 1 ml tungstate-selenate solution (1000×) (Wolin et al., 1963), 1 ml vitamin solution (1000×) (Adrian et al., 1998), 5 mM sodium acetate as carbon source and 2.5 mM 2-bromoethanesulphonate (BES) to inhibit methanogenesis. A 100 ml aliquot was dispensed in 160 ml culture flasks containing 1,2,4,5-TeCB in excess in a crystalline form (40 mg, 185 μmol). Flasks were sealed with Teflon faced rubber septa (13 mm diameter, Wheaton) and aluminum crimps, the medium was then flushed with N_2_/CO_2_ (4:1) for 25 min. Hydrogen was supplied at ∼20% of the headspace volume. Ti(III) citrate (1 mM) was supplied as the chemical reductant. Cultures were inoculated with groundwater (1% v/v) from a contaminated aquifer located near Botany Bay, Sydney, Australia and incubated at 30°C statically in the dark.

After four transfers (1% v/v), dilution-to-extinction experiments were performed four times in bicarbonate-free media where Ti(III) citrate was replaced by amorphous FeS (1 mM) as the chemical reductant. The medium was buffered with 3-(N-morpholino)-propanesulfonic acid (MOPS) and sparged with N_2_ instead of N_2_/CO_2_. Once strain TeCB1 was successfully isolated, incubation conditions reverted to bicarbonate medium with Ti(III) citrate and without BES. Cultures were set up in triplicate and incubated at 30°C.

### Electron Acceptor and Donor Range Tests

Cultures were performed in triplicate in bicarbonate buffered medium amended with one of the test organochlorines. Partially water-soluble organochlorines were added at a final concentration of 80μM: tetrachloroethene (PCE), trichloroethene (TCE), 1,1,2,2-tetrachloroethane (TeCA), 1,1,2- and 1,1,1-trichloroethane (TCA), 1,1- and 1,2-dichloroethane (DCA), 1,2,4-TCB, trichloromethane (TCM), dichloromethane (DCM), carbon tetrachloride (CT), 1,2- and 1,3-DCB and MCB. Other chlorinated compounds with lower water solubility were added in excess, 40 mg for solids: HCB, pentachlorobenzene (PCB), 1,2,3,4- and 1,2,3,5-TeCB, 1,2,3- and 1,3,5-TCB, 1,4-DCB, 2,4,6-trichlorophenol (TCP), hexachloroethane (HCA) and 1 μl for HCBD. The capability of strain TeCB1 to use alternative electron donors was tested by replacing H_2_ with 5 mM methanol, ethanol, formate or lactate, respectively, using 1,2,4,5-TeCB as the terminal electron acceptor.

### Organochlorine Analysis

To quantify 1,2,4,5-TeCB degradation products, 1 ml of culture was transferred to a 10 ml GC-headspace vial, samples were analyzed using a Shimadzu GC-2010 Plus Gas chromatograph (GC) equipped with a J&W Agilent DB-5 column (30m × 0.32 (i.d.) × 0.32 μm film thickness), a headspace autosampler (PAL LHS2-xt-Shim) and a flame ionization detector (FID). Prior to injection (250 μl), samples were incubated at 80°C with agitation for 2 min. The GC inlet (split ratio of 1:10) and detector temperature were maintained at 250°C, the oven temperature was held at 100°C for 1 min, then ramped at 25°C min^-1^ to 250°C and held isothermally for 0.5 min. Organochlorines added in excess were not quantified with this method. Aliphatic organochlorines, with the exception of HCBD and HCA (which were added in excess), were analyzed by manual headspace analysis directly from culture flasks where 100 ml samples were withdrawn with a pressure-lockable gas-tight syringe (SGE-Analytical Science). Samples were analyzed using an Agilent 6890N GC-FID fitted with an Agilent J&W GS-Gas-Pro column [60 m × 0.32 mm (i.d.)]. The inlet (split ratio of 1:10) and detector maintained at 250°C. The oven temperature was initially held at 80°C for 1 min and then ramped at 15°C min^-1^ to 250°C where it was held isothermally for 1 min. Aqueous chlorinated benzene standards were prepared with the same gas to liquid space ration as the cultures flasks accounting for phase partitioning according to Henry’s Law. The amount of chloride released was calculated by multiplying the concentration of daughter products (mmole l^-1^) by the difference in Cl^-^ number between the parent and the daughter compounds.

### DNA Extraction and 16S rRNA Gene PCR

Strain TeCB1 DNA was extracted from 300 ml of culture according to a previously described protocol (Murray et al., 1998). DNA concentration was determined using a Qubit^®^ 2.0 fluorometer (Life Technologies) according to the manufacturer’s instructions. Nearly full-length 16S rRNA gene PCR was performed using universal bacterial primers 8F and 1492R, amplification was conducted under the following conditions: 2 min at 95°C, followed by 30 cycles of 0.3 min at 94°C, 0.3 min at 61°C, 1.3 min at 72°C ending with an extension step at 72°C for 3 min.

### 454-Pyrosequencing

To identify the microbial community in the parent enrichment, the extracted DNA was pyrosequenced using 454-FLX (Dowd et al., 2008) using universal primers 926F (5′-AAA CTY AAA KGA ATT GRC GG-3′) and 1392R (5′-ACG GGC GGT GTG TRC-3′) (Matsuki et al., 2002). 16S PCR amplicons were sequenced using 454-FLX pyrosequencing at the Research and Testing Laboratory (Lubbock, TX, USA).

The resulting sequence reads were quality filtered, taxonomically classified and clustered into operational taxonomic units (OTUs) using MOTHUR (Schloss et al., 2009) and the associated 454 standard operating procedure. Briefly, reads shorter than 250 base pairs were discarded, aligned against a reference 16S alignment (Silva SEED), taxonomically classified using the SILVA taxonomic outlines with a 80% confidence threshold, and clustering into OTUs at 97% identity using the average neighbor algorithm. The resulting OTU by sample data matrix was used for downstream analyses.

### Denaturing Gradient Gel Electrophoresis (DGGE)

Microbial community fingerprinting was performed via DGGE targeting the V3 region of the 16S rRNA gene according to a previously described procedure (Muyzer et al., 1993) using the universal primers GC338F (5′ CGCCCGCCGCGCCCCCGCCCCGGCCCGCCGCCCCCGCCCACTCCTACGGGAGGCAGC-3′) and 530R (5′-GTATTACCGCGGCTGCTG-3′). Electrophoresis was performed with a denaturing gradient of 30–60% at 75 V, 60°C for 16.5 h.

### Clone Library

A nearly full-length 16S rRNA gene clone library of strain TeCB1 was generated using a pGEM^®^-T Easy Vector System (Promega), 16S rRNA gene amplicons were ligated into plasmids, transformed into *Escherichia coli* JM109 High Efficiency Competent cells and then screened for positive transformations. Twenty-five clones were selected and sequenced using primers T7 (5′-TAATACGACTCACTATAGGG-3′) and SP6 (5′-ATTCTATAGTGTCACCTAAAT-3′). Obtained sequences were then aligned on Geneious R9 assembly software (Kearse et al., 2012) and analyzed with BLAST-n.

### Real-time Quantitative Polymerase Chain Reaction (qPCR)

Quantitative PCR was used to estimate the concentrations of *Dehalobacter* and total bacterial population using universal bacterial primers 1048F/1194R (Horz et al., 2005) and 445F/1248R for *Dehalobacter* (Bunge and Lechner, 2001).

Reaction mixtures, 10 μl final volume, contained 5 μl of SsoFast Eva Green Supermix (Bio-Rad), 100 nM reverse and forward primers each, 2 μl of DNA template, 0.1 mg of Bovine serum albumin (BSA) (Thermo Fisher Scientific). For total bacteria quantification, cycling conditions using a CFX96 Real-Time System (Bio-Rad) were as follows: 3 min at 98°C, 39 cycles of 0.2 min at 95°C and 0.5 min at 62°C followed by melting curve analysis from 60 to 99°C. For *Dehalobacter*: 3 min at 98°C, 44 cycles of 0.2 min at 94°C and 0.45 min at 58°C followed by melting curve analysis from 55 to 95°C. Quantification of total bacteria and *Dehalobacter* 16S rRNA gene copies was performed by analyzing serial dilution of known quantities of plasmids containing partial *Dehalobacter* spp. 16S rRNA genes.

### Transmission Electron Microscopy (TEM)

Strain TeCB1 culture (50 ml) was centrifuged at 4000 × *g*, harvested cells were fixed in 0.1 M PBS with 2.5% (w/v) glutaraldehyde. Cells were washed twice with PBS and resuspended in 50 μl of 10% (w/v) BSA. For negative staining, 10 μl of the cell suspension were placed onto a glow discharged Formvar coated 200 mesh copper grid. Staining was performed with 2% (w/v) uranyl acetate solution. Images were obtained using a FEI Tecnai G2 20 TEM equipped with a BM Eagle digital camera.

### Genome Sequencing, Assembly, and Annotation

Genomic DNA was extracted according to described previously procedure (Murray et al., 1998). DNA concentration was quantified using a Qubit^®^ 2.0 fluorometer (Life Technologies). Sequencing was performed by Novogene Bioinformatics Technology (Beijing, China) with an Illumina Hiseq sequencer. A total of 2,341,965 high-quality 100-bp pared-end reads was obtained generating 67 contigs. *De novo* assembly was carried out using SPAdes 3.6.1 standard pipeline (Bankevich et al., 2012); contigs with lengths below 1 Kb were removed (N_50_ value of 105,922 bp). Annotation of the assembled genome was conducted via NCBI Prokaryotic Genome Annotation pipeline (v3.3).

### Nucleotide Sequence Version Number

This whole-genome shotgun project has been deposited at DDB/ENA/GenBank under the version number MCHF00000000.1 and is publically available^2^.

### Preparation of Crude Protein Extracts

In an anaerobic glove box strain TeCB1 culture (300 ml) was transferred to 6 ml × 50 ml screw cap centrifuge tubes modified with PVC thread tape to enable as gas-tight seal. The tubes were centrifuged at 10000 × *g* for 20 min at 4°C. Back inside the anaerobic glove box, the supernatant was discarded and the pellets resuspended in 4 ml of the supernatant, the suspension was transferred to 2 ml × 2 ml plastic screw cap tubes fitted with a gas tight o-ring and centrifuged at 10000 × *g* 10 min. The supernatant was discarded and the pellet resuspended in 500 μl of Blue Native (native-PAGE) sample buffer (Thermo Fisher Scientific), 100 μl of 5% Digitonin (Thermo Fisher Scientific) was then added to the mix together with Lysing Matrix A (MP Biomedicals) and the samples were bead-beaten (Qiagen, TissueLyser II) at 30 Hz for 2 min followed by an ice bath for 1 min and further bead-beating for 2 more minutes. The cell lysate was centrifuged at 10000 × *g* for 10 min at 4°C. After centrifugation, the crude protein extract (supernatant) was transferred to a 1.5 ml Eppendorf tube and stored at 4°C. Protein concentrations were measured using a Pierce^TM^BCA Protein Assay Kit (Thermo Fisher Scientific) according to the manufacturer’s directions.

### Dechlorinating Activity Assay with Crude Protein Extracts

Assays were performed in 2 ml glass screw cap vials containing Ti(III) citrate (2 mM), methyl viologen (1 μM), 200 μl of the crude protein extract (∼5.06 μg total protein per vial), and 1,2,4,5-TeCB (50 mM methanolic solution) or 1,2,4-TCB (50 μM), the vials were then filled up with Tris-HCl buffer (50 mM, pH 7.4) so that there was no headspace. The vials were capped and incubated at 30°C inside an anaerobic glove box for 24 h. After incubation, the entire reaction volume (2 ml) was transferred to a 10 ml headspace flask containing anhydrous sodium sulfate (0.5 g) and 1 ml sulfuric acid (1 M). The headspace vials were analyzed by GC-FID.

### Native-PAGE and Silver Staining

Crude protein extract electrophoresis was carried out in a XCell SureLock^TM^ system (Invitrogen) according to the manufacturer. Precast 4-16% Bis-Tris gels (NativePAGE^TM^ Novex, Invitrogen) were used in this assay. A protein ladder (NativeMark, Invitrogen) was diluted 1:20 in 1× sample buffer (Thermo Fisher Scientific), this was loaded into the first lane of the gel (5 ml), 20 ml of the crude protein extract was loaded into the remaining nine lanes (∼0.5 mg total protein per lane). Electrophoresis was performed at 150 V for 60 min at room temperature in an anaerobic glove box and at 200 V for 50 min at 4°C outside the glove box. After the electrophoresis, the first three lanes, containing the ladder and two crude protein samples were excised for staining; the rest of the gel was kept in anoxic water inside the anaerobic glove box. Silver staining on the first two lanes and the molecular weight marker were performed as described previously (Mortz et al., 2001).

### In Gel Dechlorination Activity Assay

The remaining unstained lanes from the native-PAGE gel were used. Outside the anaerobic glove box an unstained gel was aligned with a gel that was silver stained in order to determine the location of proteins bands, however, no visible bands were observed; therefore the gel bands were excised arbitrarily with approximately the same distance between each section using gel extraction tips. Each band was then placed in a 2 ml crimp-cap glass amber vial and returned to the anaerobic glove box. In each vial the reaction mix contained (2 ml final volume): 1 mM Ti(III) citrate, 1 mM methyl viologen, 100 mM Tris-HCl (pH 7.4), and 50 μM 1,2,4,5-TeCB (methanolic solution) or 1,2,4-TCB. Samples were incubated for 48 h inside the anaerobic glove box at 30°C. A negative heat-killed protein control was included. Dechlorination products were quantified by GC-FID.

### Peptide Extraction from Gel Slices

Prior to nano-LC-MS/MS analysis, the silver stained lane was washed with deionized water for 5 min. Bands of interest (even if not visible) were excised from section corresponding to molecular weights between 50 and 480 kDa. Excised bands were chemically reduced with 40 ml of dithiothreitol (10 mM) in ammonium bicarbonate (50 mM) for 30 min at 37°C. The solution was discarded and 40 ml of iodoacetamide (25 mM) in ammonium bicarbonate (50 mM) were then added to the each and the mix was incubated for 30 min at 37°C. The bands were washed twice with 50 ml of acetonitrile for 10 min, then 40 ml trypsin (100 ng) in ammonium bicarbonate (20 mM) were added and incubated for 14 h at 37°C, followed by a final wash with 50 ml 1% v/v formic acid and 100 ml acetonitrile for 15 min. Extracted peptides were dried and dissolved in 10 ml 0.05% v/v heptafluorobutyric acid and 0.1% v/v formic acid.

### LC/MS/MS Peptide Analysis

Protein identification was carried out via nano-LC/MS/MS at the Bioanalytical Mass Spectrometry Facility. Digested peptides were separated via nano-LC with an Ultimate 3000 HPLC (Dionex). Samples (2.5 ml) were concentrated and desalted in a micro C18 pre-column (Dionex) with 2% v/v acetonitrile in water and 0.05% v/v TFA at 15 ml min^-1^. The pre-column was then switched (Valco 10 port valve, Dionex) in line with a fritless nano column (75 m × 10 cm) with C18 media 1.9 m, 120 Å (Dr. Maisch). Peptide elution was performed using a linear gradient of 2–36% acetonitrile in water with 0.1% v/v formic acid at 200 nl min^-1^ for 30 min. Two hundred volts were applied to low volume tip (Upchurch Scientific), the column tip was positioned approximately 0.5 cm from the heated capillary (275°C) of an Orbitrap Velos-MS (Thermo Electron). Electrospray generated positive ions and the Orbitrap was operated in a data dependent acquisition mode (DDA).

A survey scan m/z 350–1750 was acquired in the Orbitrap (resolution = 30000 at m/z 400, with an accumulation target value of 1000000 ions) with lock-mass enabled. Up to the 10 most abundant ions (>4000 counts) with charge states > 2 were sequentially isolated and fragmented within the linear ion trap using collisionally induced dissociation with and activation *q* = 0.25 and activation time of 30 ms at a target value of 3000 ions. Ions selected for MS/MS were dynamically excluded.

Mass spectra were searched against a custom database of all predicted proteins in strain TeCB1 genome via NCBI Prokaryotic Genome Annotation pipeline (v3.3) and MASCOT (v.2.3) with the following search criteria: enzyme specificity was trypsin; precursor and product tolerances were at 4 ppm ± 0.4 Da, respectively; variable modification of methionine oxidation; and one missed cleavage was allowed. The ion score significance threshold was set to 0.2 and each protein was provided with a probability based Mowse (Molecular Weight Search) score (Pappin et al., 1993).

## Results and Discussion

### Isolation of Strain TeCB1

Strain TeCB1 was isolated using ground water (5 m below surface) from an organochlorine contaminated aquifer near Botany Bay, Sydney, NSW, Australia. The contaminant profile included a mixture of aliphatic organochlorines: 1,2-DCA, TCE, PCE, and TCM, whilst chlorinated benzenes were not present.

Initial enrichments involving 1,2,4,5-TeCB showed the production 1,4-DCB (85.6 μM), 1,3-DCB (51.2 μM), and 1,2,4-TCB (45.6 μM) over 140 days. MCB and 1,2-DCB were detected in trace amounts. No dechlorination was observed in abiotic controls (Supplementary Figure S1). Precipitation of Ti hydroxide was observed indicating degradation of the citrate ligand in the chemical reductant, i.e., Ti(III) citrate.

DNA from the 1,2,4,5-TeCB degrading mixed culture was extracted and 16S rRNA gene amplicons were sequenced via 454 Pyrosequencing (Supplementary Table S1). The five most abundant genera were *Treponema, Sedimentibacter*, unclassified members of the *Synergistaceae* family and of the *Bacteroidetes* phylum and *Dehalobacter* the only recognized OHRB identified in the enrichment.

Isolation of strain TeCB1 in the enrichment culture, required elimination of other hydrogenotrophic bacteria such as homoacetogens, citrate fermenting bacteria, both of which grow faster than OHRB (Antranikian and Giffhorn, 1987; Nelson et al., 2011). Citrate fermentation has been reported in *Clostridium* species (Antranikian and Giffhorn, 1987; Sorel et al., 2001). To select against bicarbonate reducing organisms bicarbonate/CO_2_ buffer was exchanged with MOPS and N_2_, BES was supplied to inhibit methanogenic archaea and Ti(III) citrate was replaced with amorphous FeS as the chemical reducing agent to select against citrate fermenting organisms.

**FIGURE 1:**
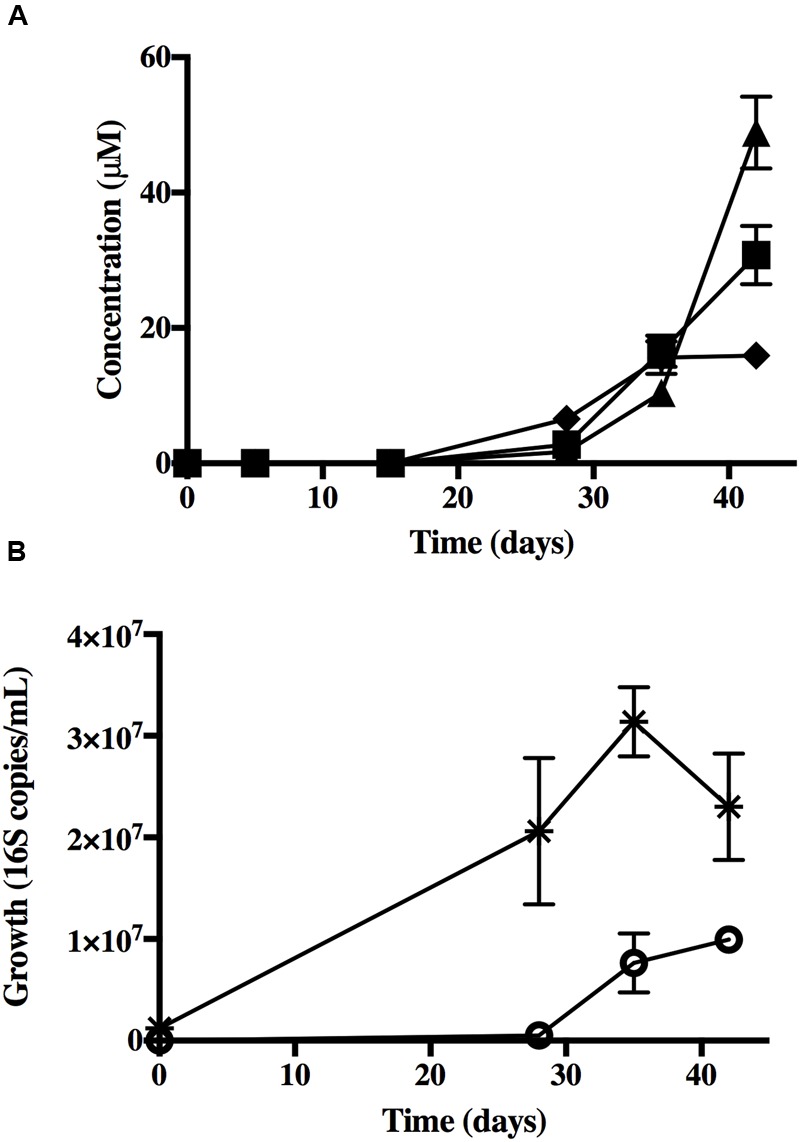
**(A)** Product accumulation in reductive dechlorination of 1,2,4,5-TeCB by a mixed culture supplied with hydrogen and acetate 1,2,4,5-TeCB was added as a crystalline form in excess and was not quantified. Dechlorination products (■) 1,3-DCB, (▲) 1,4-DCB, (◆) 1,2,4-TCB. No dechlorination was observed in the abiotic control. **(B)** Growth during the reductive dechlorination of 1,2,4,5-TeCB in the mixed culture. (✳) Total bacteria and (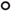) *Dehalobacter*. Error bars represent standard deviation (*n* = 3). Error bars are not visible when the standard deviation is less than size of the datum point.

The first 1% (v/v) transfer under the refined incubation conditions was used to determine that *Dehalobacter* growth was linked to the dechlorination of 1,2,4,5-TeCB, qPCR was employed using 16S rRNA universal bacterial primers and a *Dehalobacter* specific primer sets (**Figures 1A,B**) (Pappin et al., 1993). As before, the most abundant end products were 1,4-DCB (48.8 μM), 1,3-DCB (30.8 mM), and 1,2,4-TCB (19.9 mM). The initial *Dehalobacter* number of 16S rRNA copies ml^-1^ was (2.00 ± 0.66) × 10^4^, which accounted for approximately 2% of the total bacterial population at (1.23 ± 0.88) × 10^6^ 16S rRNA copies ml^-1^. After 42 days of incubation, *Dehalobacter* increased two orders of magnitude to (1.00 ± 0.10) × 10^6^ 16S rRNA copies ml^-1^ (**Figure 1B**) accounting for 30% of the total bacterial population which was (3.31 ± 1.01) × 10^7^ copies ml^-1^. The *Dehalobacter* growth yield was (5.4 ± 0.54) × 10^12^ cells mole^-1^ of chloride released. The quantified number of 16S copies ml^-1^ equates directly to cells ml^-1^ assuming that the detection of one 16S rRNA gene using RNAMER (Lagesen et al., 2007) and HMMER (Wu et al., 2011) is correct. However, it is possible that additional 16S rRNA genes remain undetected amongst the 67 contigs of the draft genome.

Four rounds of dilution-to-extinction series were performed under the refined cultivation conditions (i.e., FeS, MOPS, and N_2_) to isolate strain TeCB1. Each round was set up from 10^-2^ to 10^-8^ dilutions and incubated for 50 days (Supplementary Figure S2). Denaturing gradient gel electrophoresis (DGGE) was performed on each dilution to extinction series to monitor the microbial composition. The first dilution from the first round (10^-2^) showed the presence of only two bacteria, an uncultured *Azospira* and an uncultured *Dehalobacter* (Supplementary Figure S3). After three more rounds, where cultures were actively dechlorinating 1,2,4,5-TeCB, *Dehalobacter* sp. strain TeCB1 was finally isolated from the (10^-4^) dilution.

### Strain TeCB1 Culture Purity, Morphology, and Phylogeny

Several lines of evidence suggest that attempts to isolate strain TeCB1 were successful. Microscopic examination of strain TeCB1 cells by light and transmission electron microscopy revealed morphological features consistent with previous reports for *Dehalobacter* cells (Holliger et al., 1998; Wong et al., 2016). The strain appeared microscopically pure as rod shaped cells, occurring singly or in pairs, with a single polar flagellum. The average cell length was determined to be 1–1.5 μm and with a diameter of approximately 0.5 μm (Supplementary Figure S4).

When medium of the original composition [i.e., bicarbonate, Ti(III) citrate and N_2_/CO_2_] was inoculated with strain TeCB1, no growth was observed in organochlorine free controls showing the absence of citrate fermenting, and bicarbonate reducing bacteria. Additionally, no growth was observed in aerobic or anaerobic organic carbon rich medium (LB) inoculated with strain TeCB1 culture, suggesting the absence of readily cultivable, non-fastidious microorganisms.

**FIGURE 2:**
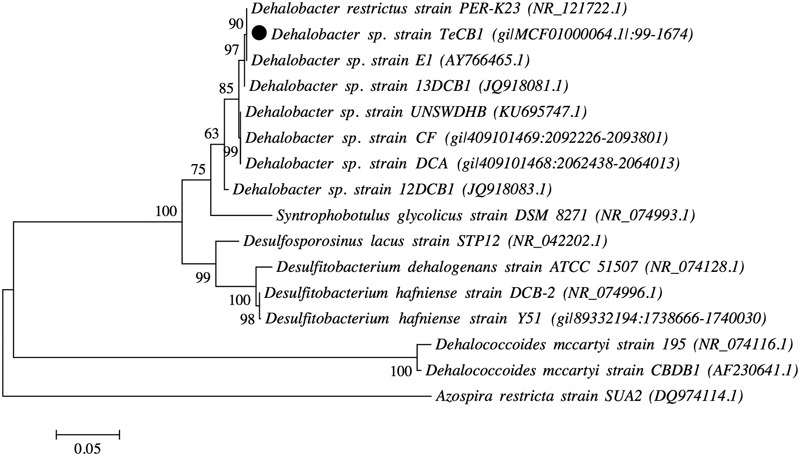
**Maximum likelihood 16S rRNA gene tree of *Dehalobacter* sp. strain TeCB1, along with other *Dehalobacter* strains, OHRB and bacterial species.** Sequence alignment and tree construction were performed with MEGAv.6 (Tamura et al., 2013). Numbers adjacent to tree branches represent percentage of branch support based on 1000 bootstrap re-sampling. Scale bar shows an evolutionary distance 0.05 nucleotide substitutions per site. Numbers adjacent to bacterium names indicate GenBank accession numbers, the project version and the location of 16S rRNA gene within the genome. *Dehalococcoides mccartyi* (strains CBDB1 and 195) and *Azospira restricta* strain SUA2 were used as outgroups.

Clone libraries (16S rRNA) were constructed; the purified amplicons from the clone libraries were then profiled by DGGE (Supplementary Figure S5). All 25 clones showed a single band with the same migration distance. Sequencing revealed the closest relative to all clones was *Dehalobacter restrictus* strain PER-K23 (Query cover 99%, Identity 99%). Near full-length 16S rRNA gene sequences were obtained from five clones, in Supplementary Figure S6 the full 16S sequence obtained from the annotated genome is shown. All five sequences were ∼1380–1410 base pairs long and most closely related to strain PER-K23 (Query cover 100%, Identity 99%). The total genomic comparison provided by NCBI genome neighbor report where the gapped identity of strains PER-K23 and TeCB1 is 99.1% (Supplementary Table S2). A maximum likelihood phylogenetic tree was constructed including all known *Dehalobacter* strains able to use chlorinated benzenes, phenols or chlorinated aliphatics as electron acceptors (**Figure 2**). Strain TeCB1 was clustered with *Dehalobacter restrictus* PER-K23 and E1.

### General Genomic Features

Genomic annotation revealed that strain TeCB1’s full genome is 3.13 Mb long, with a GC content of 44% and contains 2962 coding sequences (CDS), 50 tRNAs and one detectable 16S RNA gene copy (Alfan-Guzman et al., 2017) (Supplementary Table S3). Strain TeCB1 encodes for 23 full-length and one truncated RDase homologs (*rdhA*) (Supplementary Table S4), five of which are unique to strain TeCB1. A genome quality assessment tool (CheckM) was used to assess the quality and purity of the genome (Parks et al., 2015), it reported a completeness of 99.94% based on the finding of 418/420 lineage specific marker genes (marker lineage Clostridia), contamination of 0.17% and strain heterogeneity tested by the amino acid identity between multi-copy genes was zero. The low level of contamination further confirms the purity of the isolated strain.

### Electron Donor and Acceptor Repertoire

The ability of strain TeCB1 to use alternate electron donors was tested by replacing H_2_ with lactate, ethanol, formate, and methanol. None of these substrates supported reductive dechlorination of 1,2,4,5-TeCB, suggesting that strain TeCB1 is a strict hydrogenotroph. All isolated *Dehalobacter* strains share this trait, except for strain TCA1, which is able to use formate (Sun et al., 2002).

**FIGURE 3:**
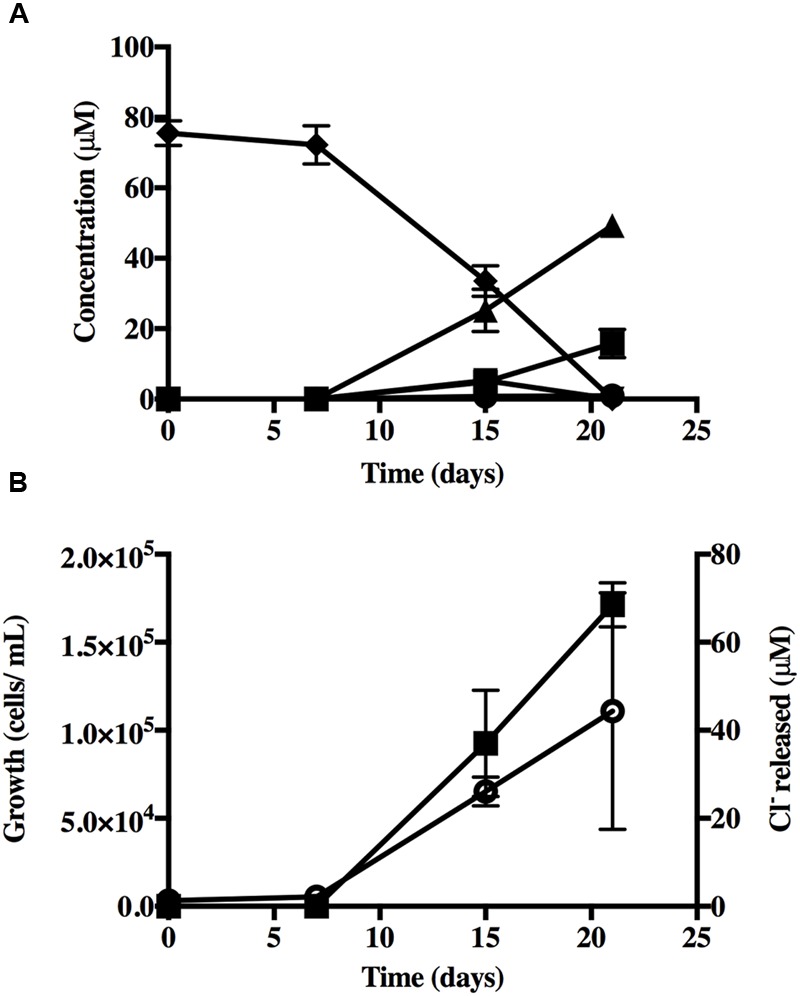
**(A)** Reductive dechlorination of 1,2,4-TCB by strain TeCB1, error bars represent standard deviation (*n* = 3). Dechlorination products (●) MCB, (▼) 1,2-DCB, (■)1,3-DCB, (▲) 1,4-DCB, (◆) 1,2,4-TCB. No dechlorination was observed in the abiotic control. **(B)** Growth determined by 16S rRNA gene qPCR and chloride released (*n* = 3) during 1,2,4-TCB respiration. (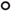) Growth and (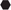) chloride released.

Of the 23 alternative organochlorines tested only1,2,4-TCB supported growth (**Figure 3** and Supplementary Table S5). 1,2,4-TCB (80 μM) was transformed to 1,4-DCB (50.7 μM) and 1,3-DCB (15.7 μM) (**Figure 3A**). Accumulation of 1,2-DCB (5.35 μM) was detected after 15 days of incubation as well as MCB (∼1.00 μM) (mass balance = 91%). However, cultures amended with 1,2-DCB did not produce MCB, suggesting that its production during 1,2,4,5-TeCB and 1,2,4-TCB respiration was co-metabolic. Growth of strain TeCB1 was monitored by qPCR of the 16S rRNA gene. Cell yield increased by 30-fold between time zero (3.0 ± 2.0) × 10^3^ and day 21 (1.10 ± 0.67) × 10^5^ 16S rRNA copies ml^-1^ (day 21, **Figure 3B**) corresponding to a yield of (1.66 ± 0.80) × 10^12^ 16S rRNA copies mole^-1^ of chloride released. Given that 1,2,4-TCB is an intermediate to dechlorination of 1,2,4,5-TeCB to dichlorobenzenes, then subtracting the growth yield on 1,2,4-TCB from that of 1,2,4,5-TeCB can approximate the growth yield due to the transformation of 1,2,4,5-TeCB to 1,2,4-TCB (i.e., 3.74 ± 0.98) × 10^12^ 16S rRNA copies mole^-1^ chloride released.

**FIGURE 4:**
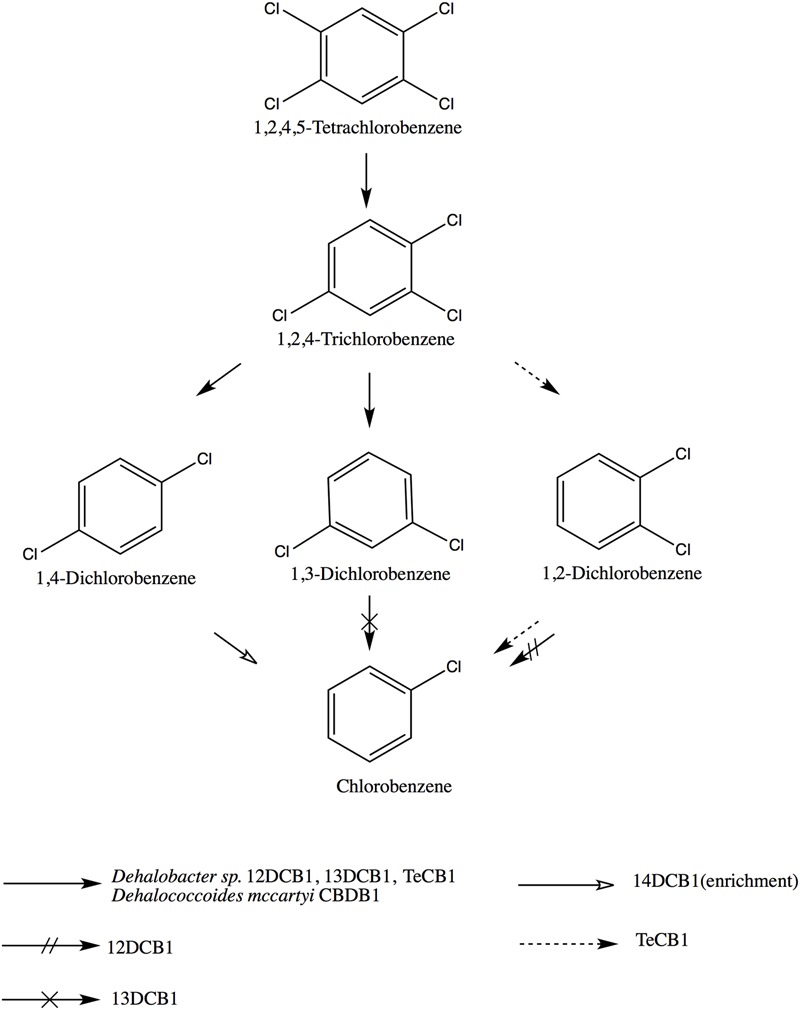
**Reductive dechlorination of 1,2,4,5-TeCB by different OHRB, including: *Dehalobacter* sp. strain TeCB1 and *Dehalobacter* sp. strains 12DCB1, 13DCB1, 14DCB1 (Nelson et al., 2014), and *Dehalococcoides mccartyi* CBDB1 (Adrian et al., 2000).** Note, chlorobenzene production by strain TeCB1 is co-metabolic.

Other *Dehalobacter* strains capable of 1,2,4,5-TeCB and 1,2,4-TCB respiration have been described (strains 13DCB1, 12DCB1, and 14DCB1) (Nelson et al., 2014). Despite being able to respire the same substrates the four strains (including TeCB1) show different product profiles (**Figure 4**). All four strains transformed 1,2,4,5-TeCB to 1,2,4-TCB and then 1,3-DCB and 1,4-DCB but not 1,2-DCB, a product profile also exhibited by *D. mccartyi* strain CBDB1 (Jayachandran et al., 2003). However, only 12DCB1 and TeCB1 are able to reductively dechlorinate 1,2-DCB to MCB, showing preference for singly flanked chlorines.

Of the two isolated *Dehalobacter* strains able to respire chlorinated benzenes (12DCB1 and 13DCB1) (Nelson et al., 2014), TeCB1 is more closely related to strain 12DCB1, since their 16S rRNA genes are 99.74% identical (98.44% with 13DCB1), both strains are able to dechlorinate 1,2,4,5-TeCB and 1,2,4-TCB, however, the main product in strain 12DCB1 is MCB (Nelson et al., 2014). While strain TeCB1 seems to co-metabolically produce MCB in low levels from 1,2-DCB, its most abundant dechlorination product is 1,4-DCB.

Strains PER-K23 and TeCB1 share 99% identity in their 16S rRNA gene (which is complete), however, strain TeCB1 possesses only a single detectable copy of this gene, while PER-K23 has four (Supplementary Table S3). The genome of strain TeCB1 encodes for 24 predicted reductive dehalogenases (RdhA), one of them N-terminally truncated, while strain PER-K23 genome contains 20 full-length RdhA orthologs and four truncated (Kruse et al., 2013) (Supplementary Table S4).

Strain TeCB1 was unable to respire PCE or TCE, a feature also observed in one of its closest relatives, strain E1 (van Doesburg et al., 2005). The other chlorobenzene respiring *Dehalobacter* sp. strains 12DCB1 and 13DCB1 are able to use PCE and/or TCE (Nelson et al., 2014). However, genomic information for strains 12-, 13- and 14DCB1 are currently unavailable, therefore genomic comparisons are not possible.

### Identification of the 1,2,4,5-TeCB Reductive Dehalogenase (TcbA)

**FIGURE 5:**
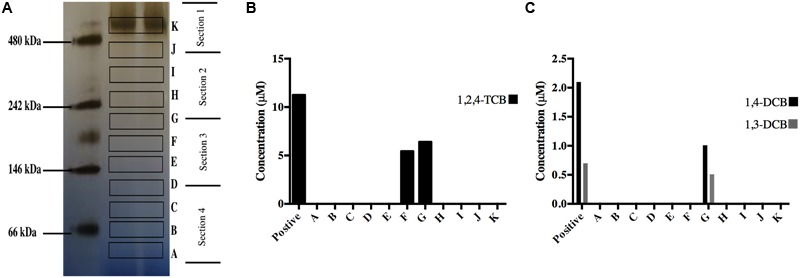
**(A)** Native-PAGE gel with molecular weight marker (left lane), silver stain samples (center lane) and lettered segments indicating gel slices excised and subjected to dechlorinating activity assays (right lane) (Sections on the right side of the gel indicate how a full lane was excise for LC/MS/MS analysis). Dechlorinating activity assay using eluted proteins from unstained excised gel slices with either 1,2,4,5-TeCB **(B)** or 1,2,4-TCB **(C)**, in both cases the shown concentrations correspond to the dechlorination end products. The positive control was performed using the crude protein extract.

Silver stained native-PAGE gels only showed a single band close to the 480 kDa region, therefore gel bands were excised arbitrarily with approximately the same distance between each section, bands A to K (**Figure 5A**). To obtain sufficient dechlorinating activity eight slices from eight unstained lanes at the same position (A to K) were pooled into a single test to increase the overall protein concentration in the assay. Reductive dechlorination of 1,2,4,5-TeCB was detected in slices F and G (∼200 and 250 kDa, respectively). The assay containing 1,2,4-TCB showed activity only in band G, where 1,3 and 1,4-DCB were the main products, 1,2-DCB was detected in trace amounts, no MCB was observed (**Figures 5B,C**). A crude protein extract in suspension was used as positive control, while a heat-treated extract was used as negative control where dechlorination was not detected.

In gel assays with 1,2,4,5-TeCB as a substrate, the sum of dechlorination products (bands F and G) matched up to what was obtained in the positive control (**Figure 5B**). On the other hand in the assay containing 1,2,4-TCB, dechlorinating activity was at least twofold lower than in the positive control, bearing in mind that activity assays were performed on gel slices pooled from eight lanes. The positive control (crude protein extract) represents the potential activity in one lane. The diminished activity in gel slices is probably due to oxygen exposure, as the bands were excised outside the anaerobic glove box. The effects of oxygen of RDases has been previously reported, where dechlorinating activity was completely lost after 128 h of exposure of crude protein extracts from *Desulfitobacterium hafniense* PCP-1 to air (Bisaillon et al., 2010).

In crude protein extracts using 1,2,4,5-TeCB or 1,2,4-TCB as substrates, the main dechlorination products were 1,2,4-TCB and 1,3- and 1,4-DCB. In contrast, live cultures of strain TeCB1 were able to produce quantifiable amounts of 1,2-DCB that decreased as MCB was produced. The absence of MCB in the crude protein extracts and in the in gel activity tests strengthens the argument that its production in living cultures is co-metabolic. Co-metabolic production of lesser-chlorinated compounds has been described in other OHRB, e.g., the reduction of vinyl chloride to ethene by *D. mccartyi* 195 (Maymo-Gatell et al., 1999).

LC/MS/MS analysis of the gel slices revealed that only one of the 24 predicted RdhAs was found in slices shown to have reductive dechlorination activity (i.e., slices F and G). The protein corresponds with gene locus tag A7D23_RS01445, now named *tcbA*. A full lane of the gel (divided in four sections as shown in **Figure 5**) and a crude protein extract were also analyzed by LC/MS/MS, TcbA peptide signals were detected in all samples except for section 1 of the quartered gel (Supplementary Table S6). In total the amino acid sequence coverage of TcbA was 29.40% (Supplementary Figure S7). Additionally, MS peptide signals were detected for two other RdhAs in low abundance (locus tag A7D23_08165 and 14460), the amino acid sequence coverage of these proteins was <3% with emPAI scores < 0.07 (Supplementary Table S6). Neither of them was detected in the actively dechlorinating gel slices F and G; therefore it is concluded that TcbA was responsible for the reductive dechlorination of 1,2,4,5-TeCB and 1,2,4-TCB.

The detection of multiple RdhAs in OHRBs grown with single substrates has been observed before. In pure cultures of *D. mccartyi* CBDB1 grown with 2,3-dichlorophenol as sole electron acceptor, a PceA ortholog (cbdbA1588) was detected as the most abundant RdhA, along with two other RdhAs (cbdbA80 and cbdbA88) in lower abundance (Morris et al., 2007). It has been hypothesize that expression of certain RdhAs maybe broadly induced by the presence of halogenated substrates or could be constitutively expressed (Adrian et al., 2007). The expression of two additional *rdhA*s in strain TeCB1 during growth with 1,2,4,5-TeCB and 1,2,4-TCB requires further investigation.

The predicted amino acid sequence showed that the molecular weight of TcbA is 61.5 kDA. However, the gel bands with dechlorinating activity were positioned between the 240 and 146-kDa region (**Figure 5A**). This suggests that TcbA might exist in a protein complex. It has been shown that PceA is a dimeric RDase in *Sulfurospirillum multivorans* (Bommer et al., 2014) and a recent report showed the chlorinated benzene reductase (CbrA) in *D. mccartyi* CBDB1 is part of an eight-protein complex (Kublik et al., 2015).

**FIGURE 6:**
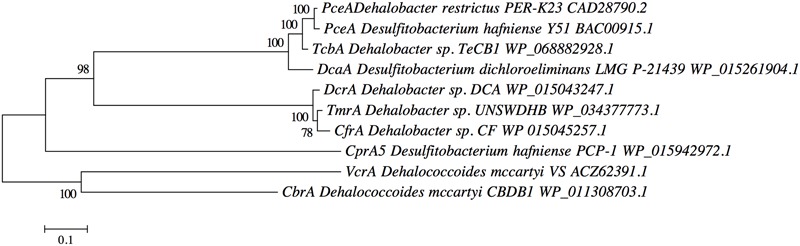
**Neighbor-joining tree of TcbA in *Dehalobacter* sp. strain TeCB1 along with other OHRBs RDases.** All RdhAs in the tree are shown with their NCBI version numbers. Sequence alignment was generated using the MUSCLE algorithm and tree construction were performed with MEGA6: Molecular Evolutionary Genetics Analysis v.6 (Tamura et al., 2013). Numbers next to branches indicate percentage of branch support based on 1000 bootstrap re-sampling. The scale bar indicates an evolutionary distance of 0.1 nucleotide substitutions per site.

TcbA is encoded by 1656 nucleotides transcribed into 551 amino acids. The TcbA amino acid sequence was aligned with CbrA (Adrian et al., 2007), the only chlorobenzene RDase identified to date, and were shown to have low identity (19%) (**Figure 6**). CbrA was found in *D. mccartyi* strain CBDB1 and catalyzes the reduction of 1,2,3,4-TeCB to 1,2,4-TCB. Surprisingly, TcbA shares 95 and 94.5% identity with PceA from *Dehalobacter restrictus* PER-K23 and *Desulfitobacterium hafniense* Y51. Protein alignment of TcbA to PceA(s) of PER-K23 and Y51 revealed 10 amino acid substitutions (Supplementary Figure S8). It has been shown that even a single amino acid substitution may affect substrate selection or orientation (Marsavelski et al., 2014). A RDase classification system based on pairwise amino acid identity (>90%) has recently been proposed (Hug et al., 2013), under this criteria, TcbA would fall into Group 6, which contains other RdhA from other OHRB like *Dehalobacter restrictus* PER-K23, WL, CF and from *Desulfitobacterium hafniense* Y51 and *Desulfitobacterium dichloroeliminans*.

Typical consensus motifs found in all RDases were found in the predicted sequence of TcbA including a Tat signal which is involved in transport across the membrane and two 4Fe-4S clusters, involved in electron transfer (Supplementary Figure S8). Similar to other RDases, e.g., strain PER-K23 (PceA) or UNSWDHB (TmrA) (Kruse et al., 2013; Wong et al., 2016) no corrinoid-binding consensus sequence (DXHXXGSXLGG) (Hug et al., 2013) was observed in TcbA. However, possible corrinoid-binding motifs close to the C-terminus in DcpA of *Dehalogenimonas lykanthroporepellens* strain BL-DC-9 have been identified (Padilla-Crespo et al., 2014). The lack of these sequences could indicate that the insertion of the cobalamin moiety relies on other binding motifs (Maillard et al., 2003; Futagami et al., 2008).

### TcbA Associated Proteins and Configuration of the *tcbA* Gene Cluster

The anchor membrane protein with three transmembrane helices, TcbB (A7D23_RS01440), was not found by mass spectrometry. RdhB peptides are notoriously difficult to be detected by LC/MS/MS involving trypsin digestion because of the limited number of cleavage sites and because of the difficulty in detecting small and low abundance hydrophobic peptides produced (Olsen et al., 2004; Adrian et al., 2007; Morris et al., 2007; Tran et al., 2011; Tang et al., 2012).

Similarly, TcbC (A7D23_RS01435) and TcbT (A7D23_RS01430) were not detected either by LC/MS/MS. However, the genes encoding for these proteins related to transcription regulation of RdhA (*rdhC*) and the chaperone (*rdhT*) involved in the correct folding of the catalytic subunit A (RdhA) precursor protein during the Tat secretion process were found in the annotated genome. In strain TeCB1 these genes are located in the same gene cluster as *tcbA* and *tcbB* (Supplementary Figure S9). This configuration is also found in PceA from PER-K23 and *Desulfitobacterium hafniense* strain TCE1 (Maillard et al., 2005; Duret et al., 2012). In other cases, the *rdhT*, e.g., in strain UNSWDHB, it is outside the *rdhA* gene cluster (Jugder et al., 2015, 2016a; Wong et al., 2016).

### Other Proteins Detected by LC/MS/MS

Other proteins of interest were detected via LC/MS/MS (Supplementary Table S6). Among these, two commonly found stress-induced chaperones in proteomic assays during the anaerobic biodegradation of organochlorines (Smidt et al., 2000; Adrian et al., 2007; Tang et al., 2013), DnaK (A7D23_RS09795) and GroEL (A7D23_RS01130), the latter is involved in the prevention of protein aggregation and facilitation of protein folding, it has also been suggested that it could also be involved in the assembly and cofactor insertion during the maturation of RR signal peptide-containing proteins like RDases (Smidt et al., 2000). GroEL overexpression was observed in *Desulfitobacterium hafniense* TCE1 when TCE was used as final electron acceptor (Prat et al., 2011).

Strain TeCB1 like all other *Dehalobacter* strains was shown to use hydrogen as the electron donor. As with strain PER-K23 (Kruse et al., 2013; Rupakula et al., 2013), strain TeCB1 possesses nine different hydrogenases, including two six-subunit, membrane-bound energy-conserving Ni/Fe hydrogenases (one Ech-type), three Ni/Fe periplasmic membrane-bound uptake hydrogenases (Hup-type), three iron only hydrogenases and one formate dehydrogenase (Supplementary Table S7). Additionally, all hydrogenase subunit gene copy numbers are identical in strains TeCB1 and PER-K23 (Supplementary Table S7).

LC/MS/MS detected the presence of two subunits of one of the uptake hydrogenases, HyaA (A7D23_RS03765) and HyaB (A7D23_RS03765) (Supplementary Table S6). The complete set of genes for menaquinone biosynthesis was also identified in strain TeCB1 (*menA*BCDEFGHI) (A7D23_RS15030, 15035, 15040, 15045, 15050, 15055, 15060, and 03135).

Other enzymes involved in the energetic metabolism were also detected; electrons transported from hydrogenases to RDases lead to the generation of proton motive force which results in ATP formation by transmembrane ATPases (Stroo et al., 2012). LC/MS/MS showed presence of ATPase subunits alpha and beta (A7D23_RS0230 and 02920) and the F1F0 ATPase synthase subunit alpha (A7D23_RS029235), the latter has been shown to play an important role in the energy generation of *D. mccartyi* CBDB1 and 195 (Nijenhuis and Zinder, 2005; Stroo et al., 2012).

### Annotated Genes for Cobalamin Biosynthesis and the Wood-Ljungdahl Pathway

Genomic annotation revealed a full complement of genes for cobalamin metabolism including cobalamin *de novo* biosynthesis, cobinamide salvaging pathway and cobalt and corrinoid transporters (Supplementary Table S7). Like strain PER-K23, strain TeCB1 contains gene encoding two distinct cobalt transport systems namely *cbi*MNQO and *cbi*MNQ, the latter system is unique to strains PER-K23 (Rupakula et al., 2014) and TeCB1. Genes encoding for corrin ring biosynthesis or modification (Goris et al., 2015) are also found in strain TeCB1’s genome (*cbiA, cbiD, cbiE, cibG, cbiJ*) of these, only CbiE (A7D23_RS03170) was detected in the proteomic analysis. BtuF (A7D23_RS07670), a corrinoid ABC transporter, was also found via LC/MS/MS.

To date all sequenced *Dehalobacter* genomes contain a full complement of cobalamin biosynthetic genes (Rupakula et al., 2014; Wong et al., 2016; Wang et al., 2017). However, strain PER-K23 has a truncated *cbiH* gene that was hypothesized to render this strain incapable of cobalamin biosynthesis, and therefore reliant on exogenous cobalamin supply (Rupakula et al., 2014). This hypothesis was further validated when strains UNSWDHB and CF, possessing a full-length *cbiH* gene (locus tag DCF50_RS14480 and UNSWDHB_RS14695, respectively) were shown to grow without exogenous cobalamin supply (Wong et al., 2016; Wang et al., 2017). Despite TeCB1 being closely related to strains PER-K23 and E1 (also incapable of *de novo* cobalamin biosynthesis), it possesses a full-length *cbiH* gene (A7D23_RS 03205), which could confer it the capability of growth without exogenous addition of cobalamins, however, this hypothesis needs experimental confirmation.

TeCB1 also possesses a complete set of genes encoding the Wood-Ljungdahl pathway (WLP) (Supplementary Table S7). Complete or partial WLP encoding genes have been reported in other OHRB, including: *Dehalobacter restrictus* strains PER-K23 and UNSWDHB (Kruse et al., 2013; Rupakula et al., 2013; Jugder et al., 2016b; Wong et al., 2016) among others. The WLP pathway can function in either reductive or oxidative directions. In the reductive direction bicarbonate is reduced to formate and then ultimately to acetate used in biomass production via acetyl-CoA. In the oxidative direction acetate can be oxidized to bicarbonate thus releasing electron for electron acceptor reduction (Schauder et al., 1988). Interestingly, given that strain TeCB1 was isolated and grown in bicarbonate-free medium, this suggests that its growth is independent of the reductive direction of the WLP.

## Conclusion

*Dehalobacter* sp. strain TeCB1 was successfully isolated and characterized, this OHRB is able to use 1,2,4,5-TeCB and 1,2,4-TCB as terminal electron acceptors with the production of 1,3 and 1.4-DCB, and co-metabolic production of MCB from 1,2-DCB.

The RDase (TcbA) responsible for the reductive dechlorination of both terminal electron acceptors was functionally identified, and is the first chlorinated benzene RDase characterization outside the genus *Dehalococcoides*. TcbA showed a strikingly high level of identity with PCE RDase (PceA, 94% amino acid identity) from strain PER-K23 than to the only other chlorinated benzene RDase functionally characterized (CbrA, 30% amino acid identity) from *Dehalococcoides*. This finding shows that a small number of amino acid substitutions can have a large effect on substrate specificity. This is an encouraging finding for those who seek to manipulate RDase substrate specificity via site-directed mutagenesis in heterologous expression systems. Additionally, the identification TcbA broadens the capacity of bioremediation practitioners and alike to detect *in situ* chlorinated benzene degradation through qPCR primer specifically designed to target *tcbA*.

## Author Contributions

RA-G: Conception, design, analysis and execution of the experimental work, manuscript drafting. HE: data analysis and interpretation, manuscript drafting. MM: conception, design, data analysis and interpretation, manuscript drafting. ML: conception, design, data analysis and interpretation, manuscript drafting.

## Conflict of Interest Statement

The authors declare that the research was conducted in the absence of any commercial or financial relationships that could be construed as a potential conflict of interest.

## Footnotes

1 https://archive.epa.gov/epawaste/hazard/wastemin/web/pdf/tetchlben.pdf

2 https://www.ncbi.nlm.nih.gov/nuccore/1055168950
